# Degrees of change: the promise of anti-racist assessment

**DOI:** 10.3389/fsoc.2023.972036

**Published:** 2023-10-06

**Authors:** Melissa Green, Claire Malcolm

**Affiliations:** ^1^Faculty of Wellbeing, Education and Language Studies, School of Education, Childhood and Youth Studies, The Open University, Milton Keynes, United Kingdom; ^2^Faculty of Arts and Social Sciences, School of Social Sciences and Global Studies, The Open University, Milton Keynes, United Kingdom

**Keywords:** higher education, whiteness, neoliberalism, degree awarding gaps, racial inequity, anti-racism, anti-racist assessment, critical race theory in education

## Abstract

Assessment practices in Higher Education remain beholden to the twin pillars of neoliberal economic orthodoxy and White supremacy. The former has given rise to the modularization and commodification of education, wherein student performance is measured according to narrow and often meaningless metrics that foster and maintain ineffective assessment mechanisms. The latter imbues those metrics with a deference to, and valorization of, “Whiteness” as a marker of success, and this manifests in persistent awarding gaps across the sector. Critical Race Theory elucidates the ways in which the “banking model” of education and assessment is implicated in a history of colonial oppression that underpins contemporary experiences of marginalization for racially minoritized students. Furthermore, the rapid proliferation of Artificial Intelligence programs is now throwing into sharp relief the fact that traditional forms of assessment are no longer functional even on their own flawed terms. The authors argue that, at this critical juncture, Anti-Racist assessment, which not only exposes and problematizes racism itself but also embeds formative feedback, drafting, collaboration, and creativity into assessment practices, offers a practical solution that can reconceptualize ‘academic excellence’ and help to identify and support a different kind of ‘good student’, reshaping the employability agenda as a force for good and reclaiming the democratizing potential of Higher Education.

## 1. Introduction

The nature and purposes of Higher Education (HE) have long been characterized, and restricted, by the twin pillars of neo-liberalism and White supremacy. Nowhere is this more apparent than in the archaic and ineffective assessment practices that remain in place at the majority of Higher Education Institutions (HEIs), in which students are expected to partake in the “banking system” of education - a one-sided transactional model in which the educator is the depositor and the students are the depositories (Freire, 1970). Banking is reproduced by a culture of Whiteness that denies alternative narratives and histories (Hall et al., 2021). It also serves to reinforce a neoliberal framework in which elites (always monied, usually white and male) can replicate and reinforce unequal power relations by ensuring the success of those who most closely resemble them. Structural inequality, in this context, is not simply an unfortunate by-product of a system that measures the value of education in light of its profitability; rather, it sits at the core of an agenda for modularizing and commodifying education in the name of ‘employability’.

Critical Race Theory illustrates that the current assessment model in place at HEIs is a social construct that embodies specific historical, social and cultural oppression (McArthur, 2016), giving rise to a conception of the ‘good student’ as one who can make use of established academic conventions, usually the traditional long-form essay, to regurgitate the ‘knowledge’ that they have banked, thereby demonstrating their capacity to reproduce the hegemonic standards into which they have been socialized. Unsurprisingly, this favors those students who have already been conditioned to understand and respond to such expectations, whilst potentially risking the marginalization of racially minoritized students, undermining their sense of belonging, and negatively impacting their academic performance (Chang et al., 2011). This, in turn, perpetuates a cycle of oppression in which certain forms of knowledge and experience, rooted in Whiteness, are valorized at the direct expense of racially minoritized groups. Not only does this stifle any potential for learning to be transformative (Hooks, 1994), but it also actively contributes to excluding certain categories of students (Bourdieu and Passeron, 1977), hence the persistence of ‘awarding gaps’ across the sector. In contrast, Anti-Racist approaches to education call on educators and institutions to be explicit in identifying and addressing race as a matter of power and equity, acknowledging the importance of lived experience, and politicizing education to uncover and dismantle the structural roots of inequality (Naseem, 2011).

Whilst all this constitutes a compelling deontological argument against current forms of assessment, an equally persuasive instrumental case can be made for the implementation of Anti-Racist assessment. The rapid development of Artificial Intelligence (AI) enables students to fulfil the requirements of the long-form essay, even without assimilating curriculum content, and a reliance on unseen examinations as an alternative is also intrinsically problematic. Authentic, Anti-Racist assessment, which embeds formative feedback, drafting, collaboration, and creativity offers a practical solution that can reconceptualize ‘academic excellence’ and help to identify and support a different kind of ‘good student’, reshaping the ‘employability agenda’ as a force for good and reclaiming the democratizing potential of HE.

In what follows, the authors expand upon this position by unpacking the nature of, and interrelationship between, neoliberalism and Whiteness. We critique the impact of this confluence on assessment practices within HE, explore and problematize current conceptions of ‘the good student’, and propose alternatives forms of assessment drawn from the tenets of Critical Race Theory. Ultimately, we contend that only Anti-Racist assessment can transform HE into a truly liberatory experience for all.

## 2. Neoliberalism, whiteness, and the flawed nature of assessment in higher education

Assessment within HE has experienced extraordinarily little variation in decades, with the most common mechanisms for measuring ‘knowledge transfer’ taking the form of attended and online exams with multiple choice, true/false tests, and short-answer tests. Additionally, essays in the form of unseen and seen questions have traditionally been used to quantify students’ progress and understanding. This uncritical reliance on traditional forms of assessment is predicated on three fundamental beliefs: that there can be a universality of meaning as to what any grade or score represents; that it is possible to separate the goals of education from the means for their attainment; and that it is possible to disaggregate conceptions of learning into cognitive or affective or conative (Berlak et al., 1992). This approach is also imbued with the influence of two distinct but related forms of dominant discourse: neoliberalism and White supremacy.

Neoliberalism is an economic and political ideology emphasizing free markets, free trade, and individual responsibility. It has been a dominant force in global politics and economics since the late 20th century, and whilst it is beyond the scope of this paper to unpack all the consequences and implications of its vast influence, it suffices to say that it has significantly impacted HE around the world, including in the United Kingdom.

Many of the neoliberal assumptions regarding the nature of meritocracy, and the capacity of competition and privatization to improve standards and services, are intrinsically problematic and demonstrably false, and there is arguably no sector more adversely affected by their continued predominance than HE. This is because the influence of neoliberalism has changed the function of universities, transforming them into businesses, the principal purpose of which is to contribute to the country’s economic productivity. This anti-intellectualism and erosion of discourse concerning social goods (Maisuria et al., 2020) drives demands for universities to comport themselves as if they are private sector corporations. As noted by Feldman and Sandoval (2018), this has resulted in the proliferation of “performance indicators” and a “metric culture,” creating a high-stakes, hypercompetitive learning environment within and between HEIs.

Neoliberalism views education through the prism of the market and market forces and dictates the management of HEIs in light of two imperatives: profitability and the production of ‘work-ready’ graduates. However, the conception of employability is defined in terms that reflect “predatory capitalist cultural strategies, which emphasize conformity, competition, and consumerism over other ontological and axiological orientations” (Butler et al., 2019, pp. 2). In other words, measuring a student’s capabilities is a process that pits them against their peers to establish which members of the cohort are best able to assimilate and reproduce knowledge claims that reinforce the neoliberal model. This inevitably results in epistemic violence as the canon, the ‘knowledge’ with which a student must emerge, is structured within the confines of neoliberal domination. It also hones and restricts definitions of the ‘good student’ by preserving the ‘banking model’ of assessment and learning. The prevailing culture adversely affects teaching by both undermining and opposing “the emancipatory potential of higher education” (Evans, 2020, p. 574), not only hindering deeper and more democratic learning but also depoliticizing the classroom. This depoliticization is key to the “corporate university” (Webb, 2018) in which the New Public Management (NPM) approach to designing, steering, and controlling public sector organizations results in increased hierarchical control within such institutions (Busch, 2017, p. 19–20). Hierarchical control, in turn, leads to the delegitimization of dissent and to complicity in existing patriarchal, colonial and racist systems within society.

In the resulting pedagogical framework, students are viewed as receptacles for the knowledge passed to them by the educator. The litmus test for how successful this process is, is the extent to which the student emerges with a recognizably ‘good grade’ and the apparent fulfilment of pre-set learning outcomes designed to be relatively consistent across HEIs. The ‘learning’ that this engenders is, by definition, partial (in both senses of the word), with students “inclined to perform and narrate” (James et al., 2021) rather than invent and critique.

Faced with intense pressure to demonstrate financial viability and adherence to the strictures of employability, HEIs often judge that the most efficient way to produce graduates is through a focus on traditional testing mechanisms (essay and exam questions), which can be easily repurposed between cohorts, and which set an ostensibly universal standard against which all learners can be judged. Focusing predominately on summative, rather than formative, feedback also promotes affordability since it limits the amount of teaching time and resource devoted to individual students, meaning that, despite its obvious pedagogical shortcomings, the summative process, which aims to understand what the students ‘know’ in relation to the curriculum outcomes, is still largely overemphasized in HE (Crisp, 2012).

In the case of essay examinations, which are experiencing something of a renaissance in the era of AI due to the perception that they are ‘plagiarism proof’, the unseen paper versions of these often create situational experiences that do not relate to whether the student is a competent writer or has a well-developed understanding of the topic they are writing about. The student is asked to write what they ‘know’ about a topic in relation to a question without the opportunity to edit. Therefore, the essay examination results in a first-draft expression, likely not representing a fair or accurate range of a student’s writing ability or thinking skills (Davis et al., 1981). This approach to assessment persists despite ample evidence that the evaluation of learning through writing examination essays, which evolved primarily in British-influenced and European education systems (Bereiter, 2003; Murphy and Yancey, 2008), provides a metric only for ‘student performance’ (under exam conditions), and cannot be relied upon as an indicator of student learning.

Similarly, summative assessments in the form of essays rely on grading, which becomes the primary focus for both student and educator, weakening the relationship between the content and knowledge being acquired by the student, and the learning experiences themselves. Statistics from many universities show students do not check their written assignment feedback when they receive their marks (Gibbs and Simpson, 2005), meaning that opportunities for learning are lost and the obsession with grades risks undercutting arguably more valuable educational objectives, such as developing self-regulation or intrinsic motivation (Harland et al., 2015). It is perhaps unsurprising that summative feedback provided on essays (even that which is couched in terms of ‘feed-forward’) often solicits limited student engagement. If objectives, criteria, and outcomes are set before the student has begun to embark on the study of a module, the student has not been involved in the construction of the knowledge acquisition, which has instead been framed in line with neoliberal metric culture. Pre-set aims of learning demonstrate no concern for who students are and what they bring to the classroom. Without opportunities to pre-engage and contribute to the construction and discussion of marking criteria and assessment practices, the practices that are provided will likely reflect the dominant discourse of the university and, since said discourse is also steeped in Whiteness and White supremacist epistemology, this doubly disadvantages racially minoritized students.

### 2.1. Unpacking ‘whiteness’ in higher education

Just as brevity precludes an engagement with all elements of neoliberalism, it is equally impossible to evaluate all the hallmarks and manifestations of Whiteness. For the purposes of this discussion, which makes no claim to be exhaustive, the ‘norms of whiteness’ upon which analysis will focus refer to what those who are perceived to be White, and those who have a proximity to Whiteness, view as usual and standardized behaviors and values. The unspoken norms of Whiteness are expected in all communication and behavior in HE and are vaguely alluded to in its policies. ‘Academic writing’, for example, can be seen as part of a larger matrix of institutional structures that compound colonial legacies’ inequities. This normalcy is continually replicated and maintained by those who are members of the university and those who are external to it but who, given the influence of the neoliberal agenda, increasingly define what the university should be. It is normalcy informed by decades of European colonialism and imperialism (Gilborn, 2005) and used as a form of domination. Yet, as hooks (1994) states, normalizing Whiteness and Othering the racially minoritized leads those with a proximity to Whiteness to believe that “there is no representation of whiteness as terror or terrorizing” (p. 45). In other words, that the dominance of Whiteness is somehow natural and apolitical. This falsity denies the colonial and imperialist history of Whiteness. The domination of European colonizers and the creation of the university by these same colonizers has led Phipps and McDonnell (2021, p. 4) to describe the university as “the master’s house” as in Lorde’s (1984) famous quote “the master’s tools will never dismantle the master’s house.” The ‘university as the master’s house’ is a metaphor for how the institution represents sites of imperialism, nationalism and colonialism in its very existence. Educationalist Webb (2018, p. 96) describes the Western university as “the imperial university,” historically and still today ‘located within a network of state apparatuses of control, discipline, surveillance, carcerality and violence’. Here, Webb highlights precisely what ‘tools’ of control the university holds to dominate those who come into contact with it and illustrates why they are, by their nature, ‘terrorizing’.

By highlighting the alliance between the academy, state power, and state formation, with the latter two already founded on Whiteness, Webb (2018) defines Whiteness as a form of domination that endures through the current political order and neoliberal economic arrangements. Instead of an educational utopia that exists separately from these societal structures and only for the public good, the university is complicit in and synonymous with upholding these systems in its very design, and its deference to norms of Whiteness as something natural, neutral, and immutable creates and sustains White supremacy across the sector.

### 2.2. The impact of whiteness and white supremacy on defining and quantifying ‘knowledge’

The impact of White supremacist assumptions and standards on the character of assessment in HE is often underestimated. It is widely accepted that academia is classist to some degree in that, historically, academic participation and success have not tended to be associated with working class identities. However, it is still considered more contentious to assert that practices within the sector are intrinsically racist, or that they are built upon structures that discriminate based on racial and ethnic characteristics, and thereby produce further racial inequality. In reality, however, the knowledge taught and assessed in the university is steeped in White supremacy. In ‘Decolonising Methodologies’, Smith (2021, p. 2) asserts that “the pursuit of knowledge is deeply embedded in the multiple layers of imperial and colonial practices.” In Thapar- Björkert and Farahani (2019), the knower is presented as the white, Western male who embodies what is deemed to be universal and places those who are others outside of this in order to reaffirm superiority. Those who are racialized as others are positioned so as to create the “necessary condition for the continuation of colonial and epistemic violence in mainstream institutions” (Almeida, 2015, p. 81). As Almeida states, “the knower and the known cannot be independent of one another; to know is always to know on some pre-defined terms” (p. 85), and the White, male westernized knower has defined these terms. Rodriguez (2006) states that we must deconstruct Eurocentric ways (or systems) of knowing that have come to dominate our spaces, yet, within the conventions of formal academic writing, there remain stark reminders of these systems, and it is widely recognized that traditional testing (framed in terms of these conventions) restricts the ability to assess higher-order thinking skills and other essential 21st-century competencies due to the nature of the item format (Koh, 2017).

Even where institutionalized racism is acknowledged, it rarely leads to meaningful, or material, changes to assessment practices. Instead, the approaches adopted by HEIs have tended to take the form of equality and/or diversity initiatives, such as the creation of roles for EDI ‘champions’ who engage in scholarship, or the establishment of separate funds or recruitment and retention drives to encourage larger numbers of minoritized and marginalized students to attend university. Further to this, James et al. (2021) note that while HEIs often issue statements and employ these roles in moments of anti-racism “crisis” such as after the death of George Floyd in 2020, EDI champions and roles like it can often be reduced to capitalizing on Black and Brown bodies, reproducing social inequality by not actually attending to structural oppression. Extending upon the notion of “interest convergence” as delineated by Bell (1980), the institutional approaches toward addressing racism in HEIs can be seen to align closely with prevailing neoliberal ideologies. This theory suggests that advancements in racial justice occur only when they converge with the interests of the dominant group—in this case, the institutional establishment. Bell’s concept aptly captures how universities opportunistically engage with Anti-Racism initiatives, such as Equality, Diversity, and Inclusion (EDI), not as a sincere effort to enact structural changes, but more as a market-driven strategy that enhances their public image.

This problematic alignment between neoliberalism and EDI efforts underscores what Sara Ahmed describes as the “non-performativity of anti-racism” (Ahmed, 2006, p. 104). The term ‘non-performativity’ captures the inefficacy of institutional gestures that purport to be in the service of racial equality but fail to bring about substantive changes. These initiatives often adopt an instrumentalist approach, reducing EDI to a set of quantifiable metrics or targets to be achieved, rather than grappling with the complexities of structural oppression. When the spotlight on social justice wanes, these roles and initiatives are often the first to be discontinued, further evidencing their superficial commitment to true reform. In the context of assessment practices, this confluence of interests translates into a perpetuation of existing power hierarchies. The focus remains on surface-level diversity—quantitative representation—rather than qualitative changes that would critically re-evaluate assessment mechanisms. Here, we find that the institution’s commitment to Anti-Racism remains performative and serves to bolster a carefully constructed image of inclusivity and social justice engagement. As Ahmed suggests, universities “create fantasy images” of themselves (Ahmed, 2006, p. 124), perpetuating the illusion of progressiveness while obscuring the material realities of institutionalized racism. To truly move beyond this paradigm, HEIs need to disentangle Anti-Racism efforts from the neoliberal agenda and embed them in the institutional fabric, rendering them resistant to fluctuations in public sentiment. It is only through such an integrated and multi-dimensional effort, one that extends beyond EDI buzzwords and market-driven motives, that we can begin to create an equitable educational environment. This involves interrogating and overhauling the curricula, pedagogical strategies, and especially assessment mechanisms, to truly reflect an Anti-Racist and inclusive ethos.

Another approach has included HEIs heeding the call from racially minoritized students and academics to ‘decolonize the curriculum’ by actively identifying and addressing where racially minoritized theorists, scientists and academics have been removed from educational content to be replaced by their White counterparts. Whilst this is intrinsically valuable and must continue to be prioritized as a core component of Anti-Racism in HE, the impact of decolonization is limited by the fact that the assessment mechanisms used to establish if and how this ameliorated curriculum content has been assimilated remain beholden to outmoded and White supremacist academic conventions. Therefore, the authors of this paper argue that this these movements are incomplete. Puwar (2004) repeatedly states that, due to the normalization of Whiteness, it is only through contestation that the institutional and structural terrain that produces racism can be made visible. The decolonization movements provide a voice to those contesting and begin to disrupt the sense of normalcy, yet, we argue, more force is needed. Expanding on Puwar’s assertion, Joseph-Salisbury (2019) notes the significance of Critical Race Theory (CRT) being used to serve as a powerful analytical tool for deciphering how universities have been “coded” as spaces of institutionalized Whiteness. However, as this paper maintains, merely identifying these spaces is insufficient. Moreover, despite decolonization initiatives being pivotal, they tend to target only the epistemological aspects of the academy, thereby neglecting the pedagogical and evaluative domains which are equally implicated in perpetuating racial inequities.

To further unpack this, the theoretical contributions of racially minoritized scholars often remain marginalized in the academic canon due to assessment mechanisms that favor certain epistemological traditions. These assessment mechanisms, which include criteria, learning outcomes and examinations, are seldom interrogated for their intrinsic biases. These structures often replicate a form of academic gatekeeping that serves to maintain the status quo and reinforce what Bourdieu (1986) would describe as “cultural capital,” steeped in Eurocentric perspectives. Hence, it is crucial that the movement to decolonize the curriculum be accompanied by a rigorous re-evaluation of the assessment criteria themselves, as well as the pedagogical approaches used in HE. Thus, we argue that an effective contestation against the normalization of Whiteness in HEIs necessitates not merely a decolonization of the curriculum but also a radical transformation of pedagogical practices and evaluative mechanisms.

Critical Race Theory (CRT) highlights the extent to which the objectivity and neutrality purported to exist in education is socially constructed (Ladson-Billings and Tate, 1995). Rather than being founded on naturally existing customs, HE is a continuation of a colonial legacy of White supremacy and domination. With reference to the banking model, Freire (1970) describes a slave and slave master relationship, where students are not allowed to discover that they are able to educate the educator - the outcome of which is an educational system that mirrors the oppressive society. In these banker-depository, slave master–slave, oppressor-oppressed dynamics, the relationship between educator and student could not and should not develop beyond a dehumanized and reactionary one. This relationship promotes didactic approaches to teaching and learning involving lecturing, teacher-centered dissemination of information, and interactions that do not extend beyond ‘chalk and talk’ and forms of assessment that are designed only to quantify the extent to which the student can ‘parrot’ that information in essays and examinations. Consequently, students are treated as “clean slates, whose cultural and linguistic histories and everyday experiences mean little in a mechanized culture of teaching and learning, built on abstracted, fragmented, instrumentalized views of knowledge” (Darder, 2018, p. 111). This ensures that in order to succeed through existing assessment mechanisms, students for whom the ‘canon’ is alienating and excluding must deny their own individualism and authenticity and allow themselves to be molded to reflect the dominant discourses to which they are exposed. Critical Whiteness Studies (CWS), a subset of CRT, extends this argument to assert that Whiteness can be internalized by all and impacts all people in society (Aronson and Meyers, 2022, p. 39). This is linked to the normalization of Whiteness in the curriculum through the staffing structures, curriculum designs, teaching and learning. Matias and Mackey (2016) also points to CWS’ claim that White people have limited understanding of their role in oppression. This aligns with the reluctance of HEIs to acknowledge their historical foundations of Whiteness, the implications of this for racially minoritized students, and the fact that practices within the field are fabricated on the idea that hegemonic Whiteness is the norm, something which impacts assessment just as profoundly as it does course content.

In short, racism is structurally ingrained in the curriculum. Whilst assessment practices such as grading, feedback, criteria design and so on are created with intentions of neutrality, when educators treat Whiteness (and the characteristics, behaviors, and modes of expression associated with it) as neutral, and the default, students are overexposed to White-dominant perspectives not only of what knowledge is but also of how it can be measured. It is these ‘power blind’ approaches that normalize awarding gaps by viewing them as agential, as opposed to structural, as the ‘failings’ of certain groups of students to acquire knowledge, rather than the failures of HEIs to provide equal opportunities for learning to be demonstrated and showcased. This is why conceptions of the ‘good student’ are inextricably linked with Whiteness.

### 2.3. Whiteness, assessment and the ‘good student’

The authors have so far demonstrated that the combined impact of neoliberalism and Whiteness undergirds an assessment regime that is not fit for purpose. This is because current assessment mechanisms are predicated on the assumption that the knowledge we ask students to demonstrate is measurable universally. Often, this belief stems from the White-centered and majoritarian nature of what we perceive as the necessary knowledge, skills and understanding with which students of HE should leave their institutions, and this diminishes the voices and stories of minoritized groups (Solorzano and Yosso, 2002) who are on the ‘edges’ of this universality.

Those on the ‘edges’ include: those students who belong to the non-traditional groups within academia, those who have English as a second language, those students who did not follow the conventional routes of education, those who come from lower socio-economic backgrounds, or mature students “who have not been in formal education for a lengthy period of time” (Nottingham Trent University, 2013, p4). In the United Kingdom, standards and norms of language and education have been defined according to the factors of Whiteness, favoring the middle-class and monolingual English speakers. Traditional students are those who are the right age, who belong to the right cultural groups, who speak the right language, who progress straight from school or sixth form college with only a gap year to separate them from this, and who have the ‘insider knowledge’ of what is to be expected when they arrive at university.

When researching students transitioning from further education to HE, Hatt and Baxter (2003, p. 25) found that “knowing the rules of the game distinguished the A-Level entrants from the other groups”, which would seem to suggest that the privilege afforded to this group has already exposed them to the ‘hidden curriculum’ in which the ‘rules of the game’ of surviving and thriving in academia are outlined. These rules dictate that a ‘good student’ is one with good study skills, good organization, good notetaking, good attendance at taught sessions, a good grasp of ‘Academic English’ and so on. Students from marginalized communities are expected to ‘integrate’ into this definition, despite rarely having had the opportunity to develop this skillset prior to their arrival at university and almost never being provided with additional or targeted support that might enable them to do so. Those who resist the hegemonic processes of integration are placed on the margins of academia and subjected to greater social containment and labeling. Social labels like ‘disengaged’ or ‘hard-to-reach’ and social containments like ‘BAME (Black and Minority Ethnic) students’ (which collapse multiple ethnicities into a monolith, othered against the unmarked identity of Whiteness) then serve to further exclude rather than include, which was primarily their purpose.

A noteworthy consequence of this is the affirmation of Whiteness as a marker of success, something which is illustrated by the oft-cited ‘awarding gap’ that impacts racially minoritized students. Although statistics concerning this disparity have been well-rehearsed, they bear repeating in light of the clarion call for meaningful Anti-Racist forms of assessment. Whilst racially minoritized British people are generally more likely than their White British peers to go to university (Modood, 2012), they remain significantly under-represented in the United Kingdom’s most selective universities (Boliver, 2015), such as Russell Group institutions and are less likely to secure the highest degree classification awards. In the United Kingdom, 81.4% of White students are awarded undergraduate degrees classified as a first or upper second (known as ‘good degrees’). However, this figure drops to 68.0% among Black, Asian, and other racially minoritized students, equating to an awarding gap of 13.4%. This shortfall has remained largely consistent for over 25 years and has been reported for every university in the United Kingdom (Advance Higher Education, 2020), and it holds firm even when other factors have been controlled for. Despite the sector’s awareness of these disparities, “it has been noted that insufficient progress is being made to tackle structural racism and systemic inequalities [in higher education (HE)], creating unacceptable challenges and outcomes for students and colleagues who work [and learn] in the sector.” (Advance Higher Education, 2020, p. 8). Nonetheless, ‘deficit’ explanations and solutions, which centre a lack of ‘engagement’ on the part of racially minoritized students, continue to inform the sector’s response to the challenge of reducing awarding gaps.

In reality, the disparity in outcomes is attributable to several factors that are rooted in racism and White supremacy. Thomas and Quinlan (2023) note that a hostile campus environment that compromises minority students’ potential to thrive has been cited as a critical causal factor. There are discriminatory practices in teaching, learning, and student support, as well as institutional racism and a lack of understanding of how Whiteness impacts the student experience. Additionally, intrapersonal factors such as staff expectations and prejudiced attitudes associated with linguistic competence should be considered along with interpersonal factors, which may be attributed to the lack of representation in staff and students, leading to feelings of isolation or a lack of belonging. These factors promote and sustain structural inequalities (Museus, 2014; Mountford-Zimdars et al., 2015). Crucially, current assessment practices take no account of these variables, cleaving instead to false notions of neutrality and equality in the design and implementation of assessment mechanisms and representing a final, and often insurmountable, barrier for those who have already been subjected to marginalization and discrimination in every aspect of their student experience.

Having consistently failed to rise to the challenge of fostering a fairer and more inclusive student experience for racially minoritized learners, HEIs have instead created a model for the ‘good student’, which further entrenches racial bias and maintains awarding gaps. This is even more problematic in view of the proliferation of AI technology, which further throws into question the limitations of such an approach.

### 2.4. Is AI now the ‘good student’?

In late 2022, OpenAI released a new version of ChatGPT, a sophisticated natural language modeling system capable of creating unique conversational interactions while preserving and responding to the context of the discussion. The panic that ensued in HEIs regarding students using these tools was mostly, if not entirely, related to the fact that ChatGPT has the potential to disrupt traditional assessment practices in HE (Rudolph et al., 2023). ChatGPT’s ability to generate impressive prose that is indistinguishable from human-written text (and undetectable using most current forms of anti-plagiarism software) raised valid concerns about its impact on current assessment practices and the neoliberal focus on ‘rigor’ and ‘performance’. ChatGPT and other popular language models are often based on ‘machine learning’, meaning they are ‘taught’ how to process and create language, and like our HE students, these models are taught to ‘bank’ the information they are fed. That is to say that language models are codified in a way that is not dissimilar from how intellect is codified in HE. Joseph-Salisbury (2019) argues that “intellect has already been codified as white”, and the authors of this paper contend that this is likely to be further consolidated as AI models ‘trawl’ from sources that have been legitimized as canon. Furthermore, given the ways in which Whiteness and the structures that support it are normalized in HE, it should come as no surprise that AI machine learning models such as ChatGPT have been accused of racial bias and racialized stereotyping. These are systems created by humans, and thus, they often reflect the same prejudices and biases inherent in their creators. Hundt et al. (2022) provided empirical evidence of this, observing behaviors in machines that were racially stratified in ways that mimic the racism inherent in HE (and in wider society).

In the past few years, ChatGPT has been used within HE for anything from writing research grant applications to generating entire academic articles. While this has created an ‘ethical’ debate for many about whether a non-human author can be considered a contributor to the creation of knowledge (UNESCO, 2023), the key consideration for assessment practices in HE is how easily their requirements can now be fulfilled or circumvented by AI programmes. This speaks to the fallibility of assessment methods, which have encouraged students to learn by rote and regurgitate information. These models, to which students now have free access, offer an unparalleled opportunity to enquire about, create, and simulate knowledge. Furthermore, they offer a way of emulating a particular academic writing style, meaning that, for a student not already well-versed in ‘appropriate academic conventions’, these models have the potential to become a crutch. Not knowing how to ‘write like an academic’ or being unable to convey ideas in the accepted manner could lead some to rely heavily on such AI tools. More broadly, however, if developments in AI can so easily undermine assessment mechanisms in HE, the question then becomes whether they are fit for anything beyond quantifying a student’s ability to mimic a specific writing style. As such, the challenges posed by programmes such as ChatGPT are both technical and profoundly cultural and ethical. In addition to concerns about racial bias, there is a further danger that AI may perpetuate the established norms and hegemonies of academic discourse, sidelining non-traditional or marginalized voices even further. Thus, instead of democratizing access to knowledge, unchecked use of these models might further entrench academic elitism.

Thus, Rudolph et al. (2023) are correct to suggest that significant changes to traditional assessments, such as essays and online exams, are necessary to address the existence of powerful AI like ChatGPT and that universities need to adapt and update their assessment methods to incorporate the use and critique of such tools. However, the need for this shift is not merely a response to AI’s capabilities but also an imperative to challenge entrenched academic norms. For too long, assessment in HE has been dominated by hegemonic ‘academic’ techniques that do not holistically gauge a student’s true knowledge, skills, or potential. Instead, they often measure one’s adeptness at conforming to a narrow set of conventions. By mere focusing on traditional essays or exams, we risk privileging those well-practiced in these specific modes, student or machine, potentially sidelining genuine talent that might manifest differently. As such, while AI’s potential to mimic academic prose makes the need for change more urgent, it also provides a timely opportunity for universities to reconsider what they value and measure in student learning. Adopting more diverse and inclusive assessment practices can ensure that we are genuinely evaluating knowledge and skills, rather than mere adherence to a particular academic style, or perpetuating White supremacist conceptions of knowledge and knowing.

## 3. Anti-racism and the future of assessment in higher education

So far, the authors have asserted that traditional exams, essays and assignments used in HE are tools for classism, elitism, and white supremacy, with usage rules often hidden from marginalized groups. Hamstrung by the neoliberal economic agenda and its narrow definitions of employability, HE creates the ‘good student’ template by building from characteristics that perpetuate the ‘virtues’ of Whiteness and deny White privilege. Current assessment mechanisms serve only to establish the extent to which students are successfully reproducing the power dynamics into which they have been situated, by regurgitating narrow forms of ‘knowledge’, in line with established academic conventions. Addressing this profound limitation is more imperative than ever as AI’s rapid development ensures that traditional assessment practices are not even effective on their own terms given that the ‘skills’ they purport to measure can now be replicated at the touch of a button. Put simply, current assessment practices are harmful to students in ways that explain and expand existing degree awarding gaps and overcoming inequalities across the sector necessitates the kind of a fundamental reconceptualization of assessment practices, which can only be achieved through the implementation of Anti-Racist assessment.

Anti-Racist assessment is best understood in light of broader definitions of Anti-Racism and Anti-Racist Pedagogy and is related to – but distinct from - arguments for ‘Decolonizing the curriculum’. This is because the “work of Decolonizing, or building a culture of decoloniality, carries both a symbolic idea and a lived reality of the university that is neither unitary, universal, and/or linear, nor Eurocentric in its assumptions (Hall et al., 2021, p. 904). Anti-racist pedagogy focuses on the teaching and learning practices, whereas decolonization focuses on the content being taught”. Revealing the causes and consequences of the silencing and delegitimization of marginalized voices and reinstating them in their rightful place in the field of scholarship are critical components of exposing and overturning racism in HE, but such efforts can only succeed if commensurate changes are made to assessment regimes.

Kishimoto (2018, p. 541) draws from Rodriguez et al. (2013) by defining Anti-Racist pedagogy as “an organizing effort for institutional and social change that is much broader than teaching in the classroom.” In this definition, comes the affirmation that it is “not about simply incorporating racial content into courses, curriculum, and discipline. It is also about how one teaches, even in courses where race is not the subject matter. It begins with the faculty’s awareness and self-reflection of their social position and leads to the application of this analysis in their teaching, but also in their discipline, research, and departmental, university and community work” (Kishimoto, 2018, p. 541). Anti-Racist assessment, therefore, cannot be designed only with a view to quantifying and ‘grading’ student performance. Instead, it should also provide mechanisms for HEIs themselves to reflect on how effectively they are implementing and upholding standards of Anti-Racism. Given that the existing assessment regime routinely fails racially minoritized students, HEIs and the wider sector must be held accountable for their role in propagating and maintaining White supremacy. Altering assessment practices is a fundamental step toward this accountability and can be achieved by applying the principles outlined in Critical Race Theory.

### 3.1. Using critical race theory to create anti-racist assessment practices

By adopting CRT as a framework (Ladson-Billings and Tate, 1995; Yosso, 2005), it is possible to emphasize the centrality of race, racism, Whiteness and White supremacy in describing the structures and social practice of HE’s assessment regimes. The foundational tenets of CRT are as follows:

- Racism, race, and its intersections (with gender, class, etc.) are an endemic part of society.
- CRT challenges dominant frameworks and ideologies that are White-centered or White supremacist in origin.
- Scholarship works toward social justice, including the empowerment of oppressed groups and elimination of racism and poverty.
- The experiential knowledge of people of color is a legitimate way of understanding the world.
- CRT is inter- or trans-disciplinary. (Yosso et al., 2004, pp. 3–4).

This contrasts starkly with current approaches to assessment in HE in multiple ways. Firstly, whilst the T in CRT explicitly states its place as a guide and method of inquiry, it stands in opposition to the current pedagogical framework which purports to be objective and naturally existing. CRT rejects such notions of ‘neutrality’ by correctly identifying them as synonyms for Whiteness and embraces intersectionality and positionality as a foundation for teaching, learning, and assessment. It encourages us to unpack the extent to which current systems are implicated in colonial histories and white supremacy, to call into question the assumptions underlying the banking model, and to expose the structural inequalities that characterize neoliberal conceptions of employability.

Knowledge exchange, viewed through this lens, is a two-way process, in which a student’s lived experiences are treated as a legitimate basis for their own learning and as a valuable resource in the design of curricula that take aim at social justice and the empowerment of oppressed groups. Students and educators learn from one another, with agency and critical thinking skills placed at the forefront of this learning journey. There is also much which must be ‘unlearned’ in terms of the insidious influence and (un)conscious messaging about how a ‘good student’ can be defined and how academic excellence can be identified and measured. Competition is replaced with collaboration, summative assessment is de-emphasized in favor of formative feedback, creative pedagogy is deployed to amplify and legitimize marginalized voices, authority is decentered as power is redistributed in ways which benefit minoritized students who are given opportunities to engage in spaces where it is clear their contributions are welcomed and heard, and a sense of community emerges as Anti-Racist assessment practice is built around regular collaborative projects and work where everyone (including the faculty) is invested in learning together (Kishimoto, 2018). Students graduate, therefore, not with the ‘knowledge’ they will need to assimilate into the prevailing ideologies of neoliberalism and White supremacy, but rather with the tools to dismantle and disrupt them.

In conjunction with other fundamental changes to HEIs, including a focus on diversifying teaching and research staff, and decolonizing curriculum content, Anti-Racist assessment is a crucial tool for engendering radical reform to HE. At the heart of this undertaking is the explicit abandonment of false notions of neutrality, which – as this paper has demonstrated – have long functioned as a means by which to disguise and embed White privilege. In cleaving to the outmoded notion that educators teach with ‘a view from nowhere’, traditional assessment mechanisms which take aim at a student’s ability to replicate (rather than problematize) prevailing oppressive ideologies, will inevitability perpetuate and consolidate racial discrimination, hence Joseph-Salisbury and Connelly's (2021) characterization of the neoliberal-imperial-institutionally-racist-university, which they maintain must be challenged through the legitimization of counter-storytelling and the incorporation of students’ own lived experiences into their educational journey. For those contending that the more authentic assessment practices that emerge from this process undermine academic rigor, advocates of Anti-Racist assessment assert that such approaches offer a “higher level of integrity and honesty than scholarship that purports to be objective” (Joseph-Salisbury and Connelly, 2021, p. 13).

The commitment to surfacing and respecting counternarratives and contestation also necessitates overt discussion of race and racism in all aspects of the curriculum and assessment frameworks. Instead of aiming for ‘color blindness’ or seeing discussion of race as too political, we should cause our staff and students to become aware of their social positions by encouraging them to interrogate the complexities of their identities (Kishimoto, 2018, p. 548). This means normalizing both the guilt that White students can experience, and anger that many racially minoritized students experience when they acknowledge the impact of racism. By extension, it also necessitates a re-evaluation of the term ‘safe space’ in our discussions of power and authority. As noted by Kishimoto (2018), this term has been misunderstood to mean a ‘comfortable space,’ which enables avoiding discussions of White privilege or complicity with oppression. Instead, through facilitating challenging discussions and validating various emotions that arise, and reflecting this in assessment practices, we can demonstrate how sharing vulnerabilities can help deepen analysis and build relationships amongst faculty and students, who become equals in the learning process, meaning that there is no contradiction in the space being safe, particularly for those who have historically been marginalized in HE, and challenging for those whose experience of privilege has, hitherto, been uninterrogated. Only when staff and students experience the emotions related to actively resisting the status quo and acknowledging social inequalities will they feel the impetus for change. Furthermore, rejecting notions of neutrality “cultivates a more conscious and engaged form of teaching and learning and serves to break down the power dynamics that separate teachers and learners” (Joseph-Salisbury and Connelly, 2021, p. 215). In other words, discomfort is instructive because it forces both staff and students to confront their own positionality and to develop a capacity for empathy and collaboration. It also empowers students to work confidentially alongside educators, free from the ‘oppressor/oppressed’ dynamics of the traditional classroom setting.

Enabling students to reclaim their own agency is vital for designing assessment that is fit for the purpose of developing social responsibility and transferable skills. Traditional essays and unseen examinations are reverse-engineered from pre-set learning outcomes (usually straightforwardly paraphrased from subject-specific QAA Benchmarks) to which students cannot meaningfully contribute and from which comes predominately summative feedback which they often do not, or cannot, repurpose for future learning. In contrast, Anti-Racist assessment is built in collaboration with students and created with a view to emphasizing process over outcome. It also takes account of Fallows and Stevens’ (2013, p. 40) assertion that “if learning is to be transferable, assessment must be multiple in mode and context and relate to life outside” the narrow confines of the university. For instance, a more flexible learning outcome such as ‘demonstrate evidence of critical thinking’ can be interpreted by the students themselves, whom, with guidance and support from the educator, can find creative ways in which to illustrate the development of their skillset: a learning journal tracing their understanding of their relationship with the course materials in light of their own lived experience and positionality; a critical engagement with an AI-generated overview of a given topic, in which the biases of the information retrieval process itself are subjected to scrutiny; a podcast script where the object of the exercise is to render the content accessible to a non-academic audience; a report which emerges over multiple iterations in which the student’s reflection on their redrafting decisions and the rationales that informed them takes precedence over the content itself. In each of these cases, it is the student who takes ownership over their ability to design and demonstrate their learning, and their identities (multiple, intersectional, and contested) cannot be disaggregated from the work they produce. Feedback is mainly formative, and any summative feedback or final grade (should formal grading be considered necessary) moves beyond a supposedly ‘neutral’ stamp of approval from an authority figure and into a focus on reflexivity for both staff and students.

Consciously disrupting the power and authority of the educator, and reimagining ways of knowing, also enables the overturning of a particularly problematic component of the ‘hidden curriculum’, namely the persistence of ‘Academic English’ as a tool all too often weaponized against racially minoritized students. Anti-Racist assessment practices emphasize that neutrality in tone and language is as illusory as neutrality in curriculum content. As such, feelings are to be welcomed and communication not measured by how articulate it is. Collins (2002, p. 7) notes that “oppressed groups are frequently placed in the situation of being listened to only if we frame our ideas in the language that is familiar to and comfortable for a dominant group.” Questioning these expectations and the arbitrary academic conventions to which they have given rise serves to create forms of assessment that can ‘meet students where they are’ and to ensure that a learner’s capabilities are not underestimated or derailed through unnecessarily narrow marking criteria which gatekeep access to academia, undermine students’ confidence, and contribute to high drop-out rates and awarding gaps among marginalized groups.

## 4. Conclusion: who is the ‘good student’ now?

The implementation of Anti-Racist assessment practices fundamentally reshapes understandings of what constitutes a ‘good student’. This reconceptualized definition is based not around the proven ability to harness academic conventions, rooted in Whiteness, or the ultimately somewhat arbitrary capacity to respond well to examination conditions. Instead, students who have been given the opportunity to design their own assessment by means that allow them to demonstrate both personal growth and social responsibility, emerge as graduates who are: cognizant of their own privilege and positionality; emotionally literate; confident and competent in their ability to use empathy and inclusive leadership to work closely with others from a variety of backgrounds, including those who are notionally their ‘superiors’; well-versed with developing AI technologies but also keenly aware of the limitations of these programmes in terms of their potential to consolidate inequalities; capable of reflexivity and critical thinking; and - crucially - alert to political and economic injustice and minded to tackle it in their own lives and beyond.

The skillset that traditional assessment practices erroneously claim to quantify is also better served through the mechanisms of Anti-Racist assessment in that students will still need to demonstrate the capacity to work under pressure, both independently and collaboratively; to collate and present research findings; and to identify and critique subject-specific materials that are relevant to their chosen discipline. However, the learning journey will not be confined to this narrow understanding of expertise. It will, instead, imbue the student with the broad array of skills that they need to flourish in the workplace and in wider society. It will reward effort and ability, recognizing talent in a multitude of forms, and close awarding gaps that have long favored White students over their racially minoritized counterparts. It is in this respect that the neoliberal ‘employability agenda’, which has long undermined HE through its algorithmic focus on metrics and performance indicators can be repurposed with a view to empowering the kinds of graduates who can dismantle it from the inside.

The benefits of Anti-Racist assessment are not limited to students. They also positively impact educators, who continue to develop and flourish through self-reflexive practice and increased appreciation of their own power and privilege. HEIs, more broadly, can respond more effectively - in policy and practice - to their past failures in accommodating and supporting racially minoritized students, and begin to play a meaningful role in engendering social justice by uncoupling themselves from ‘metric culture’ and taking seriously aspirations for a truly Anti-Racist approach to education. Even those more motivated by instrumental than deontological concerns must surely see the value in numbering among the first HEIs to demonstrate irrefutable evidence of successfully narrowing awarding gaps.

Finally, employers will welcome into their ranks graduates whose skills, from the practical to the interpersonal, genuinely reflect the needs of a 21st-century workplace, which recognizes and celebrates the value of diverse experiences and abilities. Anti-Racist assessment, implemented alongside decolonization and diversification, gives universities the opportunity not only to vastly improve student experience but also to push-back against an agenda which has undercut their autonomy and neutered their capacity to affect social change.

It is for all these reasons that the advantages of Anti-Racist assessment practices resonate far beyond the institutions that adopt them and the racially minoritized students who rightly benefit from them. Only by dismantling HE’s interconnected systems can we move toward a genuinely inclusive and equitable HE experience.

## Author contributions

All authors listed have made a substantial, direct, and intellectual contribution to the work and approved it for publication.

## Conflict of interest

The authors declare that the research was conducted in the absence of any commercial or financial relationships that could be construed as a potential conflict of interest.

The reviewer LH-D declared a shared affiliation with the author to the handling editor at the time of review.

## Publisher’s note

All claims expressed in this article are solely those of the authors and do not necessarily represent those of their affiliated organizations, or those of the publisher, the editors and the reviewers. Any product that may be evaluated in this article, or claim that may be made by its manufacturer, is not guaranteed or endorsed by the publisher.
